# Supplementary material for: Simulation of Daily Iron Intake by Actual Diet Considering Future Trends in Wheat and Rice Biofortification, Environmental, and Dietary Factors: An Italian Case Study
Source: Nutrients. 2024 Nov 28;16(23):4097. doi: 10.3390/nu16234097 (PMC11643582; doi:10.3390/nu16234097)
Supplement: Supplementary file 1 [file nutrients-16-04097-s001.zip › nutrients-3310263-supplementary.pdf]

Table S1. Data of basic scenario, scenario 1, scenario 2, scenario 3, and scenario 4 with respect to different age groups and gender.

		Basic scenario	Scenario 1		Scenario 2		Scenario 3		Scenario 4	
		Adequacy with Respect to the Required Iron Intake Level Twenty Years from Now Because of Climate Change Effects	Iron Intake from Bio-Fortification of Wheat and Rice and Climate Changes		Iron Intake from a Shift in Whole Wheat Consumption and Climate Changes		Iron Intake from a Shift in Rice Consumption and Climate Changes		Iron Intake from Bio-Fortification, Shift Consumption, and Climate Changes	
			Iron Increase from Bio-fortification	Resulting Adequacy	Iron Increase from Shift	Resulting Adequacy	Iron Increase from Shift	Resulting Adequacy	Iron Increase From Bio-Fortification and Shift	Resulting Adequacy
MALES										
1–2	years	−1.92	0.33	−1.59	0.22	−1.70	0.02	−1.90	0.66	−1.26
3–9	years	−3.48	0.42	−3.06	0.61	−2.87	0.02	−3.46	1.28	−2.20
10–17	years	−0.66	0.69	0.03	0.80	0.14	0.04	−0.62	1.85	1.19
18–64	years	2.64	0.69	3.33	0.68	3.32	0.04	2.68	1.68	4.32
65–74	years	3.72	0.72	4.44	0.63	4.35	0.02	3.74	1.61	5.33
FEMALES										
1–2	years	−2.12	0.33	−1.79	0.17	−1.95	0.02	−2.10	0.60	−1.52
3–9	years	−4.56	0.39	−4.17	0.43	−4.13	0.05	−4.51	1.04	−3.52
10–17	years	−8.34	0.51	−7.83	0.64	−7.70	0.04	−8.30	1.44	−6.90
18–64	years	−6.94	0.51	−6.43	0.44	−6.50	0.05	−6.89	1.18	−5.76
65–74	years	−0.04	0.51	0.47	0.56	0.52	0.02	−0.02	1.30	1.26

Data are expressed as mean and mean projection.
